# Tailoring KDIGO guidelines for lupus nephritis to the real world: a stratified and cost-aware approach

**DOI:** 10.1093/ckj/sfag267

**Published:** 2026-08-12

**Authors:** Savino Sciascia, Brad Rovin, Jürgen Floege

**Affiliations:** University Center of Excellence on Nephrologic, Rheumatologic and Rare Diseases (ERK-Net, ERN Reconnet and RITA-ERN Member), University of Turin, Turin, Italy; Division of Nephrology, Department of Internal Medicine, Ohio State University, Columbus, OH, 43203, USA; Division of Nephrology, RWTH Aachen University Hospital, Pauwelstr. 30, Aachen, Germany

**Keywords:** KDIGO guidelines, lupus, lupus nephritis, systemic lupus erythematosus

## Abstract

Translating the 2024 KDIGO (Kidney Disease: Improving Global Outcomes) lupus nephritis (LN) guideline update into routine care remains challenging. While clinical trial data support early multi-agent combination therapy for nephritis to optimize kidney protection, real-world implementation is constrained by disease heterogeneity, disparities in access to advanced therapeutics, and limited incorporation of cost-effectiveness considerations. In this perspective article, we propose a pragmatic framework to adapt KDIGO recommendations to diverse healthcare settings. First, we propose a stratification model integrating clinical response kinetics, serological markers, and histological chronicity. Second, we apply implementation science frameworks to identify diagnostic and therapeutic gaps. Finally, we outline a value-based approach to evaluate the use of biologics and small molecules in terms of short- and long-term clinical outcomes and economic sustainability. Together, these strategies provide a roadmap to improve the real-world applicability of modern LN management.

## INTRODUCTION

Lupus nephritis (LN) remains one of the most severe manifestations of systemic lupus erythematosus (SLE) and is a major determinant of long-term morbidity [1]. Despite advances in immunosuppression, a substantial proportion of patients continue to accrue irreversible kidney damage, and 10%–30% progress to kidney failure within 10–15 years [2]. This persistent residual risk has driven the shift reflected in the 2024 KDIGO (Kidney Disease: Improving Global Outcomes) Clinical Practice Guideline, which endorses early multitarget therapy, including the addition of belimumab or calcineurin inhibitors (CNIs) to mycophenolate and glucocorticoids, to maximize nephron preservation [3]. Yet, translating these recommendations into routine practice remains challenging, not because of a lack of rigor in the KDIGO evidence review, but because population-level recommendations must be operationalized across highly heterogeneous patients and healthcare systems. Patients with LN differ in histological activity and chronicity, baseline kidney reserve, proteinuria trajectories, serological activity, demographic risk factors, comorbidities, adherence barriers, and access to specialist care. In parallel, availability of timely kidney biopsy, expert nephropathology, multidisciplinary LN clinics, biologics, CNIs, therapeutic monitoring, and reimbursement pathways varies considerably across regions. As a result, the same evidence-based recommendation may require different implementation strategies depending on patient-level risk and local resource constraints. [4–6]. Moreover, KDIGO’s evidence-based guidance is derived largely from controlled trial populations that do not fully reflect the demographic, socioeconomic, and comorbidity profiles of many patients seen in clinical practice [4]. A central unresolved question is therefore not whether contemporary add-on therapies can improve outcomes in LN, but how clinicians should prioritize their use in routine care. Broad escalation of treatment may expose patients with mild, low-risk, or rapidly responsive disease to unnecessary toxicity, treatment burden, and cost. Conversely, excessive restriction may delay effective therapy in patients at high risk of irreversible nephron loss [7, 8]. These tensions highlight the need for pragmatic adaptation tools that preserve fidelity to the KDIGO recommendations while supporting individualized, context-sensitive decision-making. This need for contextualization is not unique to the present framework. Similar national and regional efforts have sought to translate international glomerular disease or LN recommendations into locally applicable guidance, including Canadian commentary on KDIGO glomerular disease guidance [9], analyses of LN care in the Arabian Gulf region [10], and Spanish Society initiatives [11] addressing awareness and implementation of LN recommendations. These examples support the broader principle that evidence-based guidelines require structured adaptation to local epidemiology, drug availability, reimbursement pathways, and care-delivery models.

In this perspective article, we propose a framework for adapting the 2024 KDIGO LN recommendations to real-world practice. The framework is built around three complementary pillars: multidimensional patient stratification to identify those most likely to benefit from early treatment intensification; implementation science concepts to map diagnostic, therapeutic, and organizational barriers; and value-based assessment to incorporate long-term clinical and economic consequences into treatment decisions. The practical output of this framework is to present a the KDIGO-adapted roadmap, which is intended as an expert-informed decision-support structure rather than a validated prediction model.

### Development of the adaptation framework

Traditional guideline implementation focuses on assessing and interpreting trial evidence; however, this linear model is insufficient for a condition as heterogeneous and resource intensive as LN. We therefore adopted a three-pillar framework, stratification, implementation, and value assessment, to aid in the adaptation of the KDIGO LN guideline [8, 12, 13]. To map implementation barriers, we utilized the Consolidated Framework for Implementation Research (CFIR) [8], examining practical constraints such as biopsy access, reimbursement policies, and organizational workflows. Recognizing that high-cost therapies are central to modern LN management, we incorporated principles of value-based healthcare. Rather than proposing new therapeutic regimens, this perspective focuses on how existing evidence can be operationalized across heterogeneous healthcare settings through risk stratification, implementation monitoring, and value-based decision frameworks.

### Stratification beyond histology

#### The histological ceiling: limitations of a static classification

For over two decades, the International Society of Nephrology/Renal Pathology Society classification system has served as the bedrock of LN diagnosis and management [14]. However, relying solely on this histological framework to guide treatment in the modern era of “add-on” therapies is increasingly insufficient. Histology provides a static “snapshot” of kidney inflammation and scarring; yet, LN is a dynamic, fluctuating disease process driven by heterogeneous immunological and nonimmune pressures that vary significantly between individuals.

The rigid application of histological class often fails to capture the nuances of patient prognosis. For example, two patients with class IV diffuse proliferative nephritis may have identical biopsy activity and chronicity scores but vastly different clinical trajectories. One may respond rapidly to standard-of-care induction, while the other one may progress to refractory disease despite aggressive immunosuppression. This divergence highlights a limit to the prognostic utility of morphology alone. Vice versa, clinical and histological activity of LN may not always correlate [15, 16]. To break through this ceiling and fully operationalize the 2024 KDIGO recommendations for mycophenolate-based therapy combined with belimumab or a CNI (so called triple therapy), a multidomain KDIGO-adapted treatment approach may help clinicians integrate histological, clinical, serological, and contextual risk factors.

#### Domain I: serological dynamics and the “immunological engine”

**Figure 1:**
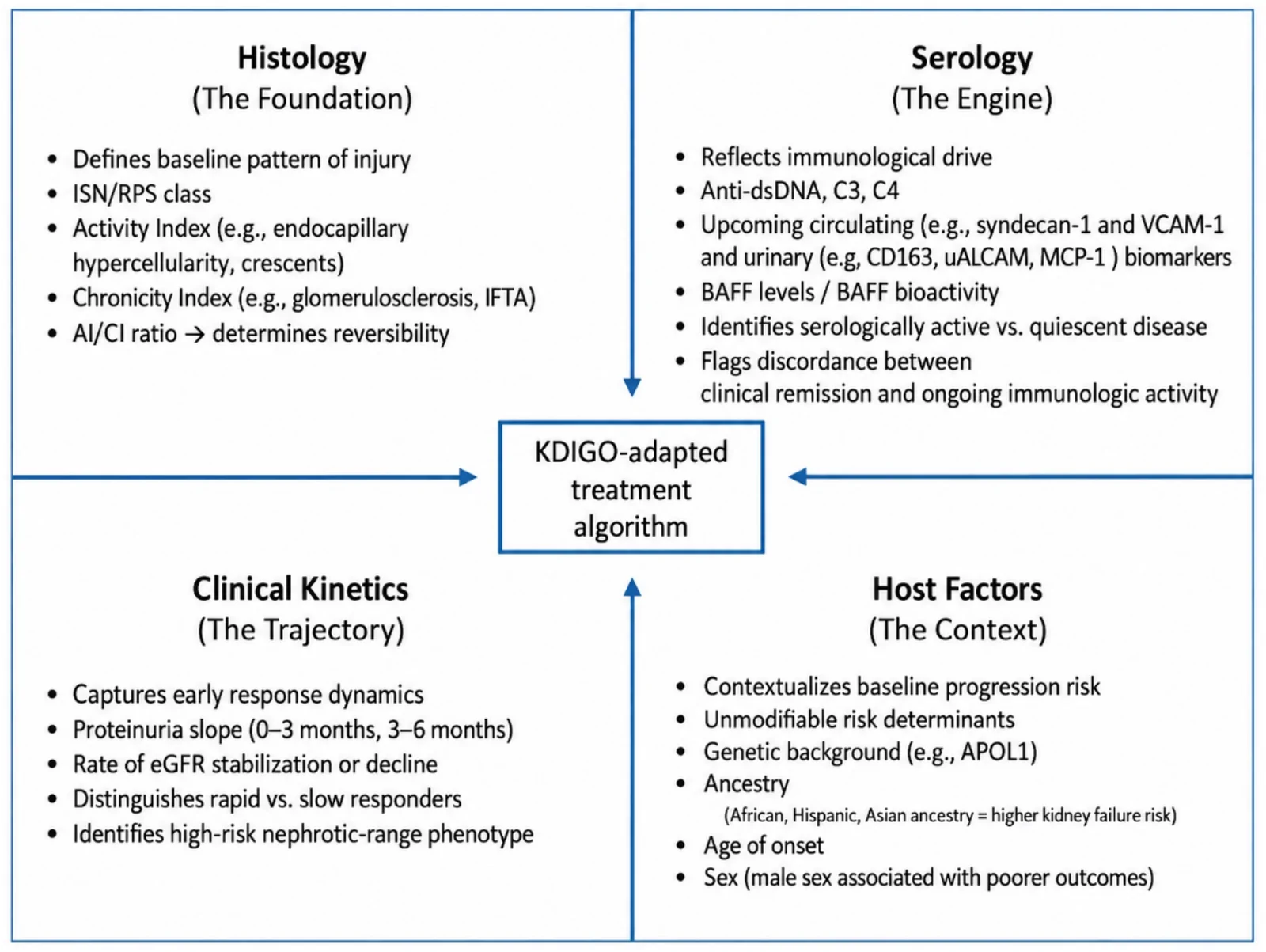
KDIGO-adapted risk stratification matrix for LN. This model illustrates the necessity of integrating four distinct domains to optimize therapeutic decision-making under the 2024 KDIGO framework. ISN/RPS, International Society of Nephrology/Renal Pathology Society; IFTA, interstitial fibrosis and tubular atrophy; anti-dsDNA, anti-dsDNA antibodies; C3, complement component 3; C4, complement component 4; VCAM-1, vascular cell adhesion molecule 1; CD163, cluster of differentiation 163; uALCAM, urinary activated leukocyte cell adhesion molecule; MCP-1, monocyte chemoattractant protein-1; BAFF, B-cell activating factor; APOL1, apolipoprotein L1.

While histology reveals the damage done, serology reflects the “engine” driving that damage (Fig. 1). A major challenge in stratification is the phenomenon of serological–clinical discordance, i.e. patients who achieve clinical remission (proteinuria <0.5 g/day) with normal glomerular filtration rate but remain serologically active [persistent hypocomplementemia and/or elevated anti-double-stranded DNA (anti-dsDNA)]. Recent longitudinal cohorts suggest these patients remain at elevated risk for “silent” histological progression and future flares [16]. For the clinician, persistent serological activity in the face of clinical response should trigger a lower threshold for maintenance therapy intensification or the addition of B-cell modulating agents like belimumab, which target the systemic autoimmune milieu. Conversely, serological normalization often precedes clinical remission, serving as an early biomarker of therapeutic efficacy that can prevent premature switching of induction agents [17].

At the same time, conventional serological markers such as complement levels and anti-dsDNA antibodies remain imperfect surrogates of intrarenal activity and do not fully capture the biological heterogeneity of LN. Emerging data suggest that broader circulating and urinary immune signatures may better reflect histologic pattern and treatment responsiveness [18]. Among others, data from the Accelerating Medicines Partnership LN cohort showed that some serum soluble mediator profiles, particularly syndecan-1 and vascular cell adhesion molecule 1, were associated with specific histologic features and response to therapy, and identified molecular subgroups not captured by histology alone. Likewise, blood immunophenotyping [19] and urinary biomarkers such as urinary activated leucocyte cell adhesion molecule, soluble CD163 and monocyte chemoattractant protein-1 [19, 20] support the concept that future stratification may benefit from integrating additional biologic signals alongside conventional serology. While a systematic appraisal of available biomarkers and their clinical utility is beyond the scope of this perspective, these examples serve to illustrate how integration of validated biomarkers may refine future risk stratification.

#### Domain II: clinical kinetics and proteinuria trajectories

The 2024 LN KDIGO guidelines emphasize the importance of achieving specific proteinuria targets (e.g. reduction by 50% at 6 months). However, this binary “pass/fail” assessment diminishes the prognostic power of response kinetics. Stratification should also distinguish between “rapid responders” (who achieve partial remission within 3 months) and “slow responders” (who exhibit a sluggish decline in proteinuria). Data from post hoc analyses of major trials (such as BLISS-LN and AURORA) suggest that the rate of proteinuria decline is a potent predictor of long-term nephron preservation [4, 23].

Patients presenting with nephrotic-range proteinuria (>3.5 g/day) represent a distinct high-risk phenotype. In these cases, the sheer volume of protein traffic across the glomerular filtration barrier exerts direct toxic effects on podocytes and tubulointerstitial cells. While overlap exists, stratification models have the potential to help in flagging these patients for therapies with direct podocyte-stabilizing effects, such as CNIs. The later are mechanistically favored to achieve rapid hemodynamic reduction of proteinuria alongside immunosuppression [4, 24].

#### Domain III: the chronicity context

**Figure 2:**
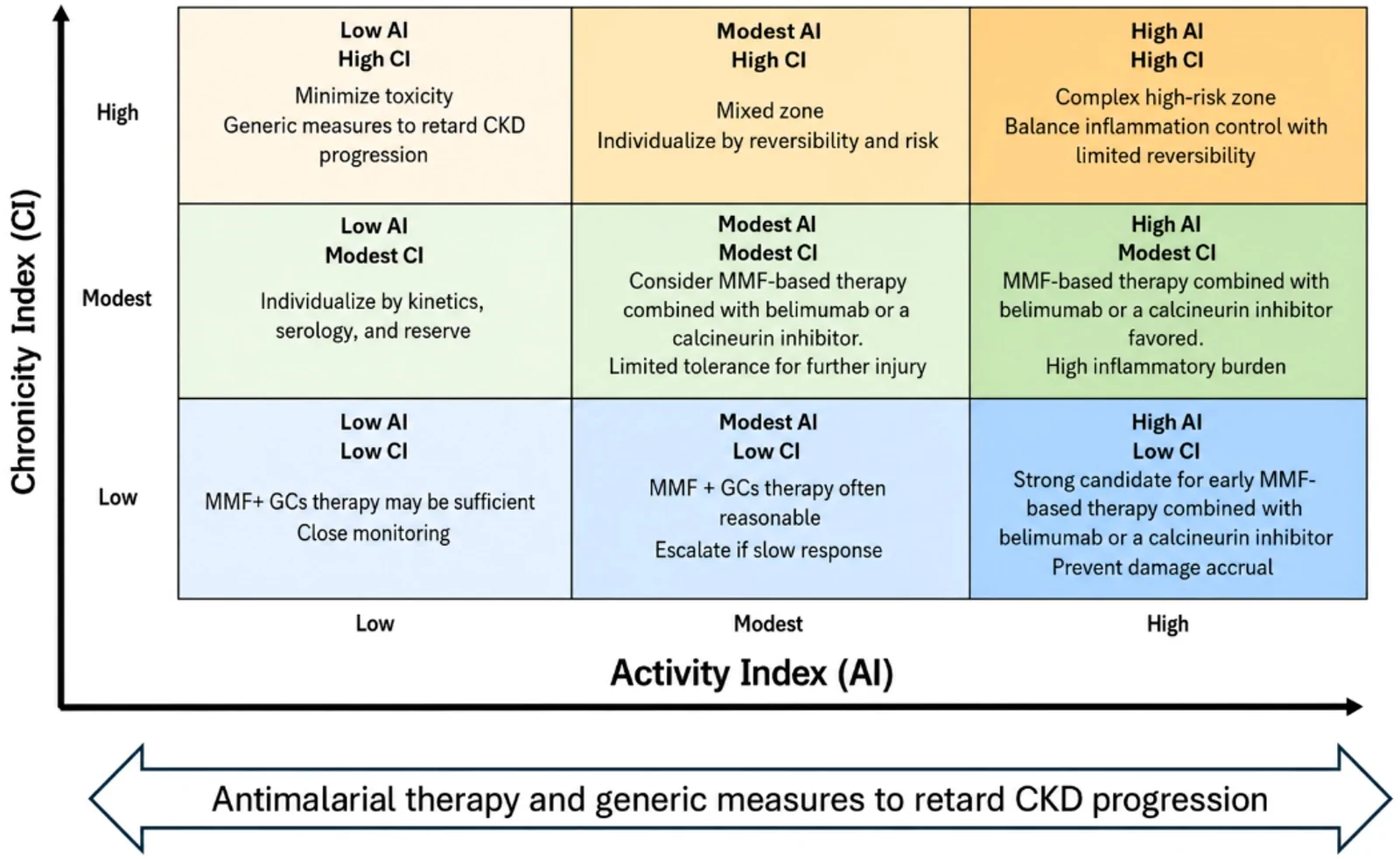
Activity–chronicity continuum for treatment adaptation in LN. This conceptual heat-map illustrates how biopsy activity and chronicity may jointly inform treatment intensity beyond histologic class alone. High activity with low chronic damage identifies patients most likely to benefit from early combination therapy. Modest activity with established chronicity may also justify treatment intensification because of limited renal reserve and reduced tolerance for further injury. In contrast, minimal activity with advanced chronicity favors a focus on generic measures to retard CKD progression and minimization of treatment toxicity. GCs, glucocorticoids.

Perhaps the most underutilized parameter in routine clinical stratification is the Chronicity Index (CI) derived from the kidney biopsy. While the Activity Index (AI) often drives the decision to initiate immunosuppression, the CI is the primary determinant of kidney reserve and reversibility. Evidence indicates that patients with a high CI (i.e. significant glomerulosclerosis, interstitial fibrosis, and tubular atrophy) have a vastly different risk–benefit ratio for aggressive immunosuppression compared to those with low CI [5, 25]. In high-CI patients, the “therapeutic window” is narrower; the kidneys are less resilient to the hemodynamic insults of LN flares, and the likelihood of recovering baseline estimated glomerular filtration rate (eGFR) is diminished. High AI with low CI represents the ideal candidate for aggressive, “add-on” mycophenolate-based therapy combined with belimumab or a CNI to prevent damage accrual [4]. At the same time, CI should not be viewed simply as an argument against treatment intensification. Rather, AI and CI define a continuum of risk. High AI with absent CI identifies patients most likely to benefit from early mycophenolate-based therapy combined with belimumab or a CNI. Modest AI with modest CI may also justify treatment intensification, because established chronic damage implies limited tolerance for further nephron loss. In contrast, modest AI with no CI may still be reasonably approached with mycophenolate-based therapy, whereas minimal or absent AI in the setting of advanced CI shifts the focus toward generic measures to retard progression of chronic kidney disease and avoidance of treatment-related toxicity. This interaction is best conceptualized as a continuum rather than a binary threshold, with treatment intensity informed by the joint distribution of activity and chronicity on biopsy (Fig. 2).

#### Domain IV: demographic and genetic pretest probability

Finally, stratification must account for the specific demographic subgroups. It is well-established that patients of African, Hispanic, and Asian ancestry face a higher risk of progression to kidney failure compared to Caucasian patients. [4] This is likely driven by genetic risk alleles (e.g. APOL1) and socioeconomic determinants of health [26, 27, 28]. As highlighted by recent large-scale cohort studies (such as the RIFLE-LN analysis), male sex and early-onset disease (pediatric/adolescent) are also independent risk factors for poor kidney outcomes. In practice, a young, male patient of African ancestry with proliferative LN starts with a fundamentally higher “pretest probability” of kidney failure than an older white female with the same histological class. Consequently, the threshold to initiate multi-agent therapy [e.g. mycophenolate mofetil (MMF) + CNI or MMF + belimumab] should be significantly lower in the former demographic to counteract this inherent biological disadvantage. However, emerging evidence may challenge our understanding of some traditional demographic risk factors for poor outcomes. For instance, a recent large international analysis showed that males and females with LN had comparable disease severity, treatment responses, kidney outcomes, and relapse-free survival [29].

In practical terms, these domains should not be interpreted as equivalent or simply additive. Histology remains the entry point for treatment adaptation, with the joint interpretation of activity and chronicity defining the amount of potentially reversible inflammatory disease and the degree of residual kidney reserve. Early clinical kinetics, particularly proteinuria trajectory and eGFR evolution over the first 3–6 months, should then be used to reassess whether the initial therapeutic strategy is adequate. Serological markers and emerging biomarkers may refine this assessment, especially in cases of clinical–serological discordance, but should not replace clinical and histological evaluation. Finally, host factors, including age at onset, ancestry-associated risk, comorbidities, adherence barriers, reproductive issues, and access to care, should be regarded as modifiers that may lower or raise the threshold for treatment intensification, but should not be used in isolation. Therefore, the proposed model is hierarchical and dynamic rather than a fixed numerical score; its weighting remains expert-informed and requires prospective validation.

### Implementation gaps in real-world LN care

Despite representing the current gold standard, the KDIGO 2024 guidelines for LN remain incompletely implemented in routine practice. We used the CFIR as an organizing lens, rather than as a formal empirical implementation study, to map barriers that may affect translation of KDIGO recommendations into daily care. In this context, implementation gaps can be grouped into outer-setting barriers, inner-setting barriers, individual-level factors, and process-related barriers.

Diagnostic barriers remain substantial. At the outer-setting level, in many low- and middle-income countries and resource-limited settings, access to specialized nephropathology is limited [4]. In addition, real-world practice often delays kidney biopsy until proteinuria is well above the recommended threshold of 0.5 g/day, potentially missing early but active disease [4]. Repeat biopsies, suggested by guidelines for refractory disease or relapse, are also inconsistently applied outside of academic centers. Therapeutic barriers are largely driven by access and cost [4, 30]. These barriers also map to the CFIR outer setting, including reimbursement policies, drug availability, and regulatory constraints. Although the guidelines recommend early use of add-on therapies such as belimumab and voclosporin in selected patients, their uptake is limited by regulatory and reimbursement constraints. In many settings, even standard therapies such as MMF remain difficult to access [30]. As a result, LN outcomes remain worse in middle-income compared with high-income countries [31]. Complex multidrug regimens may further challenge adherence if not accompanied by strong patient education and shared decision-making [32]. Similarly, organizational barriers persist within healthcare systems. These correspond to CFIR inner-setting barriers, including local workflows, availability of multidisciplinary care, access to timely biopsy, and structured follow-up pathways. Effective LN management requires close collaboration between nephrologists and rheumatologists, yet multidisciplinary care models remain uncommon. Joint clinics and structured care pathways could improve coordination, support consistent interpretation of treatment targets, and facilitate adherence to the evaluation timelines proposed in the guidelines. At the individual level, clinician familiarity with evolving LN recommendations, patient preferences, adherence, reproductive planning, comorbidities, glucocorticoid toxicity concerns, and treatment burden may further influence implementation. A recent US claims-based analysis further supports the existence of real-world implementation gaps in lupus care. Among >125 000 patients coded with lupus, diagnostic testing, biopsy use, and subspecialty involvement were inconsistent, and the authors concluded that management in the general care community did not closely follow professional society recommendations. Although broader than LN alone, these findings provide empirical support for the need to monitor and improve guideline implementation in routine practice [33].

These CFIR-informed barriers were used to shape the proposed roadmap. They highlight the need for structured risk stratification, multidisciplinary nephrology–rheumatology pathways, registry-based monitoring of implementation indicators, and context-specific value assessment. Relevant indicators may include biopsy timing, time to treatment initiation, glucocorticoid tapering, flare rates, treatment escalation in high-risk patients, and progression to chronic kidney disease or kidney failure.

### Cost-assessment and value-based care

The economic burden of LN is considerable. Real-world studies from the USA consistently show greater healthcare utilization and direct medical costs in patients with LN than in comparator SLE populations, with a further sharp escalation in costs among those who progress to advanced kidney disease or kidney failure [33–35]. Therefore, the “cost” of a new drug must be weighed against the “cost of failure,” the long-term financial burden of dialysis and transplantation [36–42].

Published cost-effectiveness analyses from the US perspective suggest that belimumab and voclosporin may fall within, or close to, commonly cited willingness-to-pay thresholds under specific modeling assumptions [41, 42]. Importantly, these estimates already incorporate downstream costs and outcomes across disease-response states, including progression to kidney failure. The main limitation is therefore not omission of averted costs, but uncertainty regarding the long-term assumptions used to extrapolate trial-based short-term benefits into lifetime renal and economic outcomes.

Real-world data from China [38] and Germany [39] consistently identify inpatient admissions as the single largest driver of direct medical costs in LN, accounting for up to 48% of total annual expenditure in the first year of diagnosis [ 38, 39]. By increasing complete renal response rates and reducing flare frequency, effective induction therapies can directly mitigate these high-cost events [39]. Also, the progression to kidney failure acts as a massive cost multiplier. In the US, the annual medical cost for a patient with LN plus kidney failure is more than double that of a patient with active LN but preserved kidney function [33, 34]. Thus, the high upfront investment in “add-on” therapies is economically justifiable if it successfully delays or prevents the transition to renal replacement therapy [40, 41].

Besides, indirect costs, specifically lost productivity and disability, constitute a further significant proportion of the total societal cost, particularly given that LN predominantly affects women of working age. Studies from Europe and North America indicate that patients with active LN have significantly higher rates of work disability and unemployment compared to controls [43–46]. In Sweden, indirect costs (productivity loss) accounted for nearly 50% of the total societal cost of SLE [47]. From a value-based care perspective, therapies that rapidly induce remission and allow patients to remain in the workforce offer a “societal return on investment” that is not captured in standard payer-perspective models. Patients with high AI, frequent flares, or demographic risk factors (e.g. young age, male sex, and African ancestry) represent the “high-value” cohort. Here, the number needed to treat to prevent one kidney failure event can be estimated to be low, justifying the cost of mycophenolate-based therapy combined with belimumab or a CNI. Conversely, for patients with mild, responsive disease (e.g. Class III with low proteinuria/chronicity), standard MMF-based therapy likely provides sufficient efficacy.

Importantly, this framework does not propose a universal cost-effectiveness threshold or a fixed cutoff at which treatment becomes justified or unjustified. Such thresholds vary substantially across healthcare systems, depending on drug acquisition costs, reimbursement policies, willingness-to-pay thresholds, dialysis and transplantation costs, and societal perspectives. Instead, stratification is proposed to increase the probability that high-cost therapies are used in patients with the greatest expected absolute renal benefit and avoided in patients in whom benefit is likely to be marginal. In an ideal setting, treatment decisions would be guided solely by expected clinical benefit, safety, and patient preferences. In real-world healthcare systems, however, cost, reimbursement, and access constraints inevitably shape implementation. Value-based adaptation should therefore aim not to restrict effective therapy, but to support equitable and sustainable access for patients most likely to derive substantial benefit. Future implementation of precision medicine may further refine this approach. Rather than promoting universal escalation, individualized biological and clinical risk assessment should help identify patients who can be managed with conventional therapy, those who are most likely to benefit from early multitarget treatment, and the small minority with severe refractory disease who may require advanced rescue strategies such as cellular therapy.

Available economic studies, including the Institute for Clinical and Economic Review assessment of belimumab and voclosporin in the USA and cost-effectiveness analyses of conventional regimens in China, support the principle that LN treatment value depends on both clinical benefit and healthcare-system context. However, no validated patient-level model currently integrates clinical, histological, economic, and health-policy variables to allocate patients to different treatment-intensity groups. Given differences in reimbursement policies, drug prices, and costs of kidney failure across countries, such a universal model may not be feasible. Accordingly, our framework should be viewed as a pragmatic value-based decision aid, not as a validated economic stratification algorithm [ 48, 49].

From this perspective, in high-resource systems, value-based care may support earlier add-on therapy for patients with active, reversible, high-risk disease. In intermediate-resource settings, cost may require more selective prioritization for patients with high histologic activity, limited chronicity, nephrotic-range proteinuria, impaired kidney reserve, recurrent flares, or inadequate early response. In resource-constrained settings, the highest-value steps may first be timely diagnosis, biopsy access, glucocorticoid minimization, blood pressure and proteinuria control, adherence support, and structured referral pathways.

The true value of a therapeutic intervention in LN is not defined by its price tag, but by its ability to alter the long-term trajectory of the disease and within the constraints of a specific healthcare system.

### Beyond the guidelines: adaptation to evolving evidence and refractory disease

While the KDIGO guidelines provide a robust starting point for treatment decisions, real-world LN care is often shaped by two challenges: the rapid evolution of the therapeutic landscape and the presence of patients with difficult-to-treat disease who do not fit standard algorithms.

A rigid adherence to guidelines in these scenarios risks therapeutic inertia. Therefore, any adaptation framework must include a dynamic component that allows for evidence-based flexibility. For instance, recent data published highlight the transformative potential of CD19-targeted Chimeric antigen receptor (CAR) T-cell therapy in achieving drug-free remission for severe, refractory autoimmune diseases [50]. Similarly, novel T-cell engagers and other cellular therapies are rapidly entering the clinic [4, 50], while recent translational studies have investigated the efficacy of intensified rituximab-based regimens [51, 52] or targeting plasma cells in refractory diseases [52, 53].

### Nonimmunosuppressive nephroprotection and cardiorenal risk reduction

Importantly, adapting KDIGO recommendations to real-world LN care should not be equated with immunosuppressive intensification alone. LN requires often simultaneous targeting of immune-mediated inflammation and chronic kidney disease that develops during/after the first episode of active nephritis. This distinction is consistent with the recently proposed concept of disease-modifying antinephropathic drugs [54], which emphasizes three complementary goals: minimizing disease activity, preventing progressive kidney structural and functional loss, and reducing treatment-related toxicity. In practical terms, all patients with LN require systematic implementation of nonimmunosuppressive nephroprotective measures, including optimized blood pressure control, renin–angiotensin–aldosterone system blockade when indicated and tolerated, reduction of residual proteinuria, glucocorticoid minimization, avoidance of nephrotoxins, smoking cessation, weight management, lipid control, and management of diabetes or steroid-induced metabolic complications.

Cardiovascular protection is particularly relevant because LN combines systemic inflammation, proteinuria, CKD, hypertension, dyslipidemia, glucocorticoid exposure, and, in selected patients, antiphospholipid antibody-related thrombotic risk. Therefore, nephroprotection and cardioprotection should be considered core components of KDIGO adaptation rather than ancillary supportive care [55]. Emerging nonimmunosuppressive therapies, including sodium–glucose cotransporter 2 inhibitors, glucagon-like peptide-1 receptor agonists, nonsteroidal mineralocorticoid receptor antagonists, and endothelin receptor antagonists, may further expand the cardiorenal protective armamentarium [4, 55]. However, LN-specific evidence remains limited, and many recommendations are still extrapolated from broader CKD or cardiovascular-risk populations. Future studies should define the optimal timing, safety, and combination of these agents in patients receiving immunosuppression for active LN.

This approach is particularly important in patients with high chronicity or limited reversible inflammatory activity, in whom the marginal benefit of further immunosuppression may be lower and the priority may shift toward slowing CKD progression, reducing cardiovascular risk, and avoiding treatment-related toxicity. Thus, the proposed KDIGO-adapted roadmap should be interpreted as a combined immunologic, nephroprotective, and cardiorenal strategy.

### Adherence as a determinant of apparent treatment failure

Treatment adherence deserves specific consideration in LN because apparent refractory disease often seems to reflect medication underexposure rather than true immunologic resistance. Nonadherence may be driven by adverse effects, access barriers, complex multidrug regimens, forgetfulness, low health literacy, limited trust, depression, reproductive concerns, or glucocorticoid toxicity. Practical strategies include shared decision-making, early discussion of expected benefits and toxicities, simplified dosing where possible, nurse- or pharmacist-led education, reminder systems, attention to medication access and reimbursement, and therapeutic drug monitoring when available, including hydroxychloroquine blood levels or mycophenolate exposure in selected cases. In the proposed roadmap, assessment of adherence should precede major treatment escalation whenever the clinical context suggests possible underexposure [56].

### A roadmap for KDIGO adaptation

**Figure 3:**
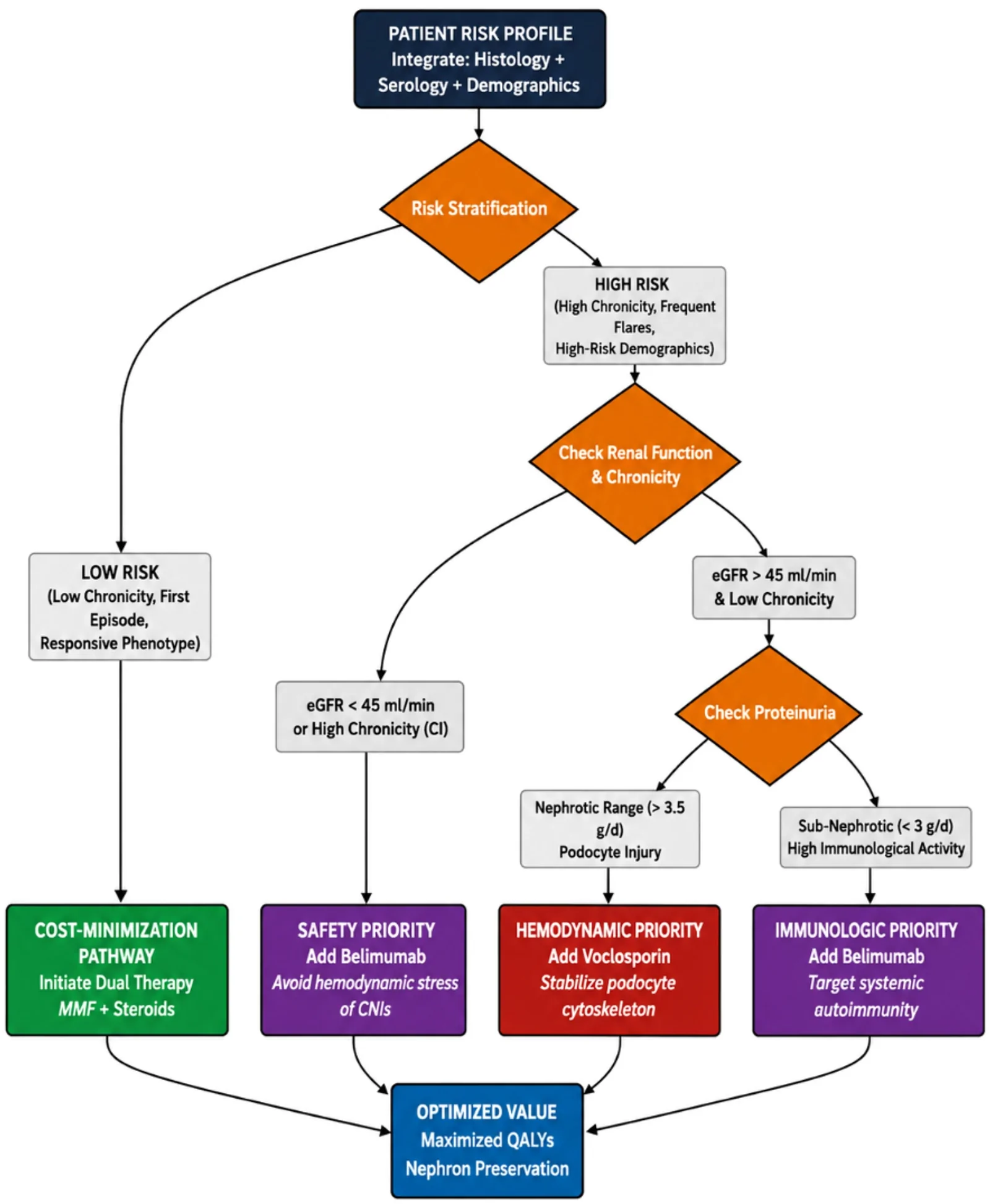
KDIGO-adapted treatment algorithm for LN. This conceptual model integrates four complementary domains, histology, serology, clinical response kinetics, and host factors, to refine therapeutic decision-making under the 2024 KDIGO framework. Histology provides the structural foundation of disease activity and chronicity; serology reflects ongoing immunological drive; clinical kinetics capture early treatment response; and host factors contextualize baseline progression risk. The intersection of these domains (“KDIGO-adapted treatment algorithm”) guides individualized treatment intensity, including selection between standard mycophenolate-based therapy and mycophenolate-based therapy combined with belimumab or a CNI in patients with active, potentially reversible, high-risk disease.

Although the KDIGO 2024 LN guidelines represent a level-setting, evidence-based starting point for the management of LN, their impact depends on effective implementation in routine care. The KDIGO-adapted roadmap presented in Figs. 2 and 3 represents the main practical outputs of this perspective article. They are intended to bridge the gap between guideline recommendations and clinical practice by translating the three proposed pillars, risk stratification, implementation monitoring, and value-based assessment, into a pragmatic decision-support structure. At the same time, the proposed roadmap is expert-informed, hypothesis-generating, and not empirically validated. Some considerations are worth mentioning. First, risk stratification should move beyond static histological classification and incorporate additional information such as clinical response over time, and emerging biomarkers. Integrating these elements into electronic health records and clinical decision support systems could support treatment selection and help clinicians tailor therapy according to disease severity and biological phenotype.

Second, implementation must be monitored through real-world data systems. Expanding LN registries would allow tracking of biopsy use, treatment patterns, and barriers to guideline adoption. These registries could serve as quality improvement tools, providing benchmarking feedback and identifying structural limitations in care delivery.

Third, many LN trials still rely on histological class and proteinuria thresholds as relatively static entry criteria, sometimes using kidney biopsies performed months or even years before randomization. This approach may misclassify patients whose proteinuria reflects chronic scarring, podocyte injury, or delayed recovery rather than ongoing active nephritis. Whenever feasible, contemporary baseline biopsy assessment should be used to document the balance between activity and chronicity and to enrich studies for patients with potentially reversible inflammatory disease. Future trials may also benefit from dynamic enrichment strategies incorporating early proteinuria trajectory, eGFR slope, serological activity, and validated urinary or circulating biomarkers, rather than relying solely on histological class and proteinuria at a single time point.

Fourth, economic sustainability must be addressed as new therapies become available. Cost-effectiveness models are needed to demonstrate that early use of effective therapies can reduce long-term complications such as kidney failure, dialysis, and transplantation. These analyses should be adapted to regional healthcare systems to inform reimbursement and policy decisions.

Because this roadmap is expert-informed and has not yet undergone formal external validation, it should be regarded as a hypothesis-generating implementation tool rather than a prescriptive treatment algorithm. Its clinical utility, feasibility, and impact on patient outcomes should be tested in prospective cohorts, registry-based studies, and pragmatic implementation studies before broad adoption. Existing infrastructures such as the European Reference Network ReCONNET, the European Rare Kidney Disease Reference Network ERKNet, and the Lupus Nephritis Trials Network provide suitable platforms to generate such real-world evidence.

## Data Availability

No new data were generated or analyzed in support of this research.
